# Supplementary material for: A Global Screen for Assembly State Changes of the Mitotic Proteome by SEC-SWATH-MS
Source: Cell Syst. 2020 Feb 26;10(2):133–155.e6. doi: 10.1016/j.cels.2020.01.001 (PMC7042714; doi:10.1016/j.cels.2020.01.001)

Q9H1E5 | TMX4_HUMAN | TMX4 KIAA1162 TXNDC13 PSEC0095 UNQ475/PRO938
Monomer MW [kDa]: 38.952 Monomer expected elution fraction: 48

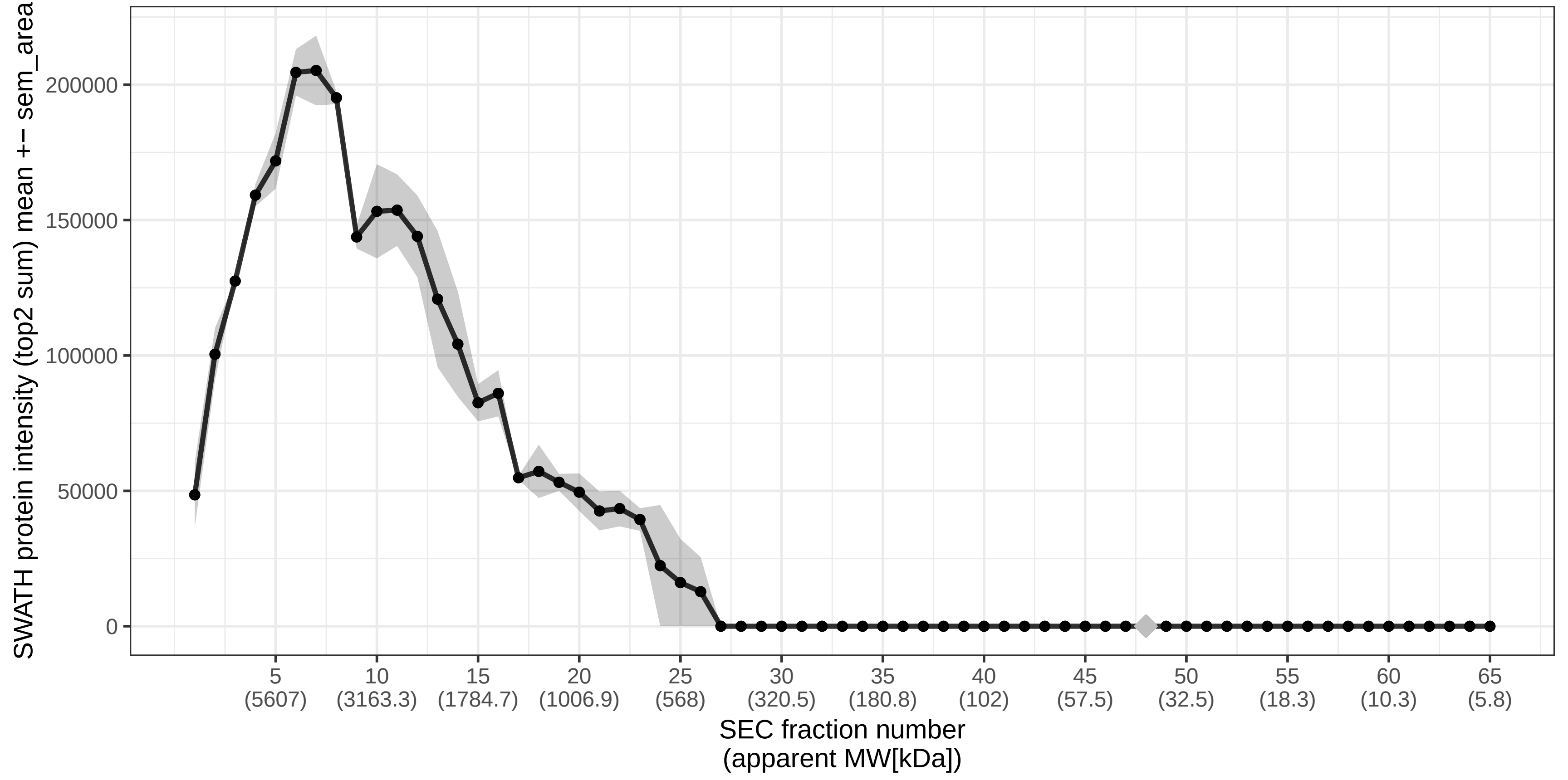

Supplement: Data S1. SEC-SWATH-MS Protein Chromatograms, Related to Figure 1 [file mmc6.zip › SECchrom_Q9H1E5_TMX4_HUMAN_TMX4_KIAA1162_TXNDC13_PSEC0095_U.pdf]
